# A cancer specific hypermethylation signature of the TERT promoter predicts biochemical relapse in prostate cancer: a retrospective cohort study

**DOI:** 10.18632/oncotarget.10639

**Published:** 2016-07-16

**Authors:** Pedro Castelo-Branco, Ricardo Leão, Tatiana Lipman, Brittany Campbell, Donghyun Lee, Aryeh Price, Cindy Zhang, Abolfazl Heidari, Derek Stephens, Stefan Boerno, Hugo Coelho, Ana Gomes, Celia Domingos, Joana D. Apolonio, Georg Schäfer, Robert G. Bristow, Michal R. Schweiger, Robert Hamilton, Alexandre Zlotta, Arnaldo Figueiredo, Helmut Klocker, Holger Sültmann, Uri Tabori

**Affiliations:** ^1^ Arthur and Sonia Labatt Brain Tumor Research Center, The Hospital for Sick Children, University of Toronto, Toronto, Ontario, Canada; ^2^ Regenerative Medicine Program, Department of Biomedical Sciences and Medicine, University of Algarve, Faro, Portugal; ^3^ Centre for Biomedical Research (CBMR), University of Algarve, Faro, Portugal; ^4^ Division of Urology, Department of Surgical Oncology Princess Margaret Cancer Center, University of Toronto, Toronto, Ontario, Canada; ^5^ Serviço de Urologia e Transplantação Renal, Centro Hospitalar Universitário Coimbra EPE, Faculty of Medicine, University of Coimbra, Coimbra, Portugal; ^6^ Sequencing Core Facility, Max Planck Institute for Molecular Genetics, Berlin, Germany; ^7^ Department of Radiation Oncology, Princess Margaret Cancer Center, Toronto, Ontario, Canada; ^8^ Department of Vertebrate Genomics, Max Planck Institute for Molecular Genetics, Berlin, Germany; ^9^ Cologne Center for Genomics, Cologne University, Cologne, Germany; ^10^ Division of Urology, Department of Surgery, Mount Sinai Hospital, Toronto, Ontario, Canada; ^11^ Department of Urology, Medical University of Innsbruck, Innsbruck, Austria; ^12^ Cancer Genome Research, German Cancer Research Center (DKFZ) and German Consortium for Translational Cancer Research (DKTK), Heidelberg, Germany

**Keywords:** TERT, prostate cancer, biomarker, diagnostic, Gleason score

## Abstract

The identification of new biomarkers to differentiate between indolent and aggressive prostate tumors is an important unmet need. We examined the role of THOR (*TERT* Hypermethylated Oncological Region) as a diagnostic and prognostic biomarker in prostate cancer (PCa).

We analyzed THOR in common cancers using genome-wide methylation arrays. Methylation status of the whole *TERT* gene in benign and malignant prostate samples was determined by MeDIP-Seq. The prognostic role of THOR in PCa was assessed by pyrosequencing on discovery and validation cohorts from patients who underwent radical prostatectomy with long-term follow-up data.

Most cancers (*n* = 3056) including PCa (*n* = 300) exhibited hypermethylation of THOR. THOR was the only region within the TERT gene that is differentially methylated between normal and malignant prostate tissue (*p* < 0.0001). Also, THOR was significantly hypermethylated in PCa when compared to paired benign tissues (*n* = 164, *p* < 0.0001). THOR hypermethylation correlated with Gleason scores and was associated with tumor invasiveness (*p* = 0.0147). Five years biochemical progression free survival (BPFS) for PCa patients in the discovery cohort was 87% (95% CI 73–100) and 65% (95% CI 52–78) for THOR non-hypermethylated and hypermethylated cancers respectively (*p* = 0.01). Similar differences in BPFS were noted in the validation cohort (*p* = 0.03). Importantly, THOR was able to predict outcome in the challenging (Gleason 6 and 7 (3 + 4)) PCa (*p* = 0.007). For this group, THOR was an independent risk factor for BPFS with a hazard-ratio of 3.685 (*p* = 0.0247). Finally, THOR hypermethylation more than doubled the risk of recurrence across all PSA levels (OR 2.5, *p* = 0.02).

## INTRODUCTION

Prostate cancer (PCa) is the most frequently diagnosed cancer and the second most common cause of cancer-related mortality among men [1]. Although one sixth of men will be diagnosed with PCa during their lifetime, only one in thirty six will die from this disease [2].

Prostate cancer is a heterogeneous disease with risk that varies according to host and tumor characteristics that have not been fully elucidated [3, 4]. In view of this heterogeneous behaviour, the clinical challenge resides in maximizing patient survival without overtreatment of indolent tumors. Despite intense research, there is a lack of validated biomarkers that can help determine the natural history of PCa. This is especially true in low risk tumors (Gleason 6) where many patients will not experience tumor progression or death from disease and some intermediate risk (Gleason 7) where tumour behavior is particularly heterogeneous [2, 4, 5].

Some patients with Gleason 6 cancers will undergo active surveillance, while the majority of patients with Gleason 7 will undergo prostatectomy or radiation therapy with curative intent. Identifying which tumors will progress to advanced disease represents a major challenge in these PCa subgroups.

Several biomarkers and algorithms which utilize multiple parameters were recently described to predict PCa behavior [6–11]. However, many of these expression and methylation signatures are too complex to provide a clinically simple and robust tool which will be useful for the treating physician. An established oncogenic process is common in most recurrent cancers and is easy to detect without complex tools and would therefore be an attractive rational target to correlate PCa progression and patient outcome.

Cancer cells achieve limitless self-renewal capacity through the activation of telomere maintenance mainly through activation of telomerase which provides immortalization for 90% of cancers [12]. The catalytic subunit of the telomerase complex is termed Telomerase Reverse Transcriptase (*TERT*) and its expression has been observed in most malignant cancers including PCa [13]. Telomere shortening and telomere length has been shown to act as a predictor of disease progression in PCa [14–16]. Moreover, telomerase activation stabilizes shorter telomeres and is a putative early marker for prostate carcinogenesis [17–22]. Analysis of telomerase activity and *TERT* expression require high quality RNA and cell extracts that are challenging, especially when paraffin embedded tissues are considered. Therefore, a DNA based assay that correlates with telomerase activity would be extremely useful as a diagnostic and prognostic tool in cancer.

Epigenetic gene regulation through DNA methylation has been associated with diagnosis and prognosis in multiple cancers including brain and prostate cancer [9–11, 23–26]. We recently identified a specific area in the *TERT* promoter, termed THOR (*TERT* Hypermethylated Oncological Region), which is hypermethylated only in cancers expressing *TERT* and non-hypermethylated in normal tissues and low-grade pediatric tumors, which do not express *TERT*. THOR predicted outcome and tumor progression in several subgroups of pediatric cancers [27].

Therefore, we postulated that THOR is hypermethylated in most cancers. In prostate cancer THOR demonstrated the diagnostic ability to differentiate cancer from normal prostate tissue and to discriminate indolent from aggressive PCa. Furthermore, THOR might be used as a marker to predict patient outcome in addition to other currently used markers.

## RESULTS

### THOR is highly methylated in common telomerase expressing cancers

**Figure 1:**
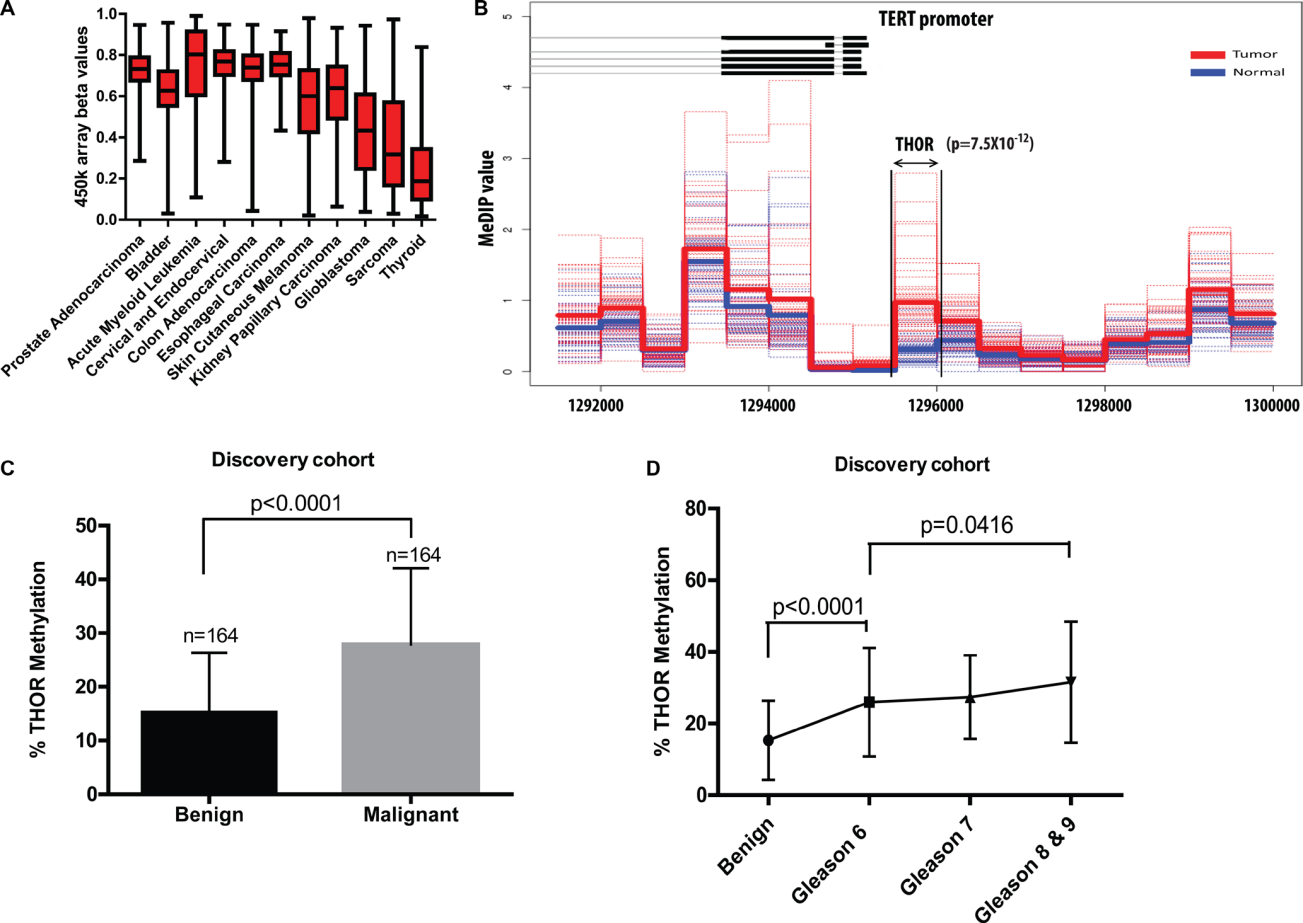
A region in the hTERT promoter (THOR) is specifically hypermethylated in malignant prostate tissues (**A**) Illumina Infinium 450 k array data obtained from the The Genome Cancer Atlas shows high THOR methylation status (cg11625005) in multiple tumors. (**B**) Methylation values of 51 tumours (dotted red lines) and their average (thick red line) as well as the methylation values of 53 normal prostate samples and their average (blue) are shown for 500 bp wide regions in the proximity of the *TERT* promoter through MeDIP-seq analysis. The methylation differences are most significant in region chr5: 1295501–1296000 (Benjamini Hochberg corrected Mann-Whitney *p*-value: 7.547178e-12) which matches the THOR. (**C**) Pyrosequencing analysis reveals that levels of THOR methylation are significantly higher in malignant prostate tissue when compared to its corresponding normals. (**D**) Levels of THOR methylation are significantly higher between any subgroup of Gleason scores and normal tissue and increase with Gleason scores, with statistical differences between Gleason 6 and Gleason ≥ 8 (*p* = 0.0416).

In order to interrogate if THOR hypermethylation is observed in common adult cancers, we analyzed the CG site within THOR (CG11625005) in 11 cancer types from the Cancer Genome Atlas (*n* = 3056, Supplementary Table S1). THOR was hypermethylated in all cancers, which rely on telomerase activation for their telomere maintenance (Figure 1A). The beta value for prostate adenocarcinoma was 0.7, indicating a high degree of methylation at THOR, albeit with a large variance. Lower and more heterogeneous methylation statuses were observed in glioma and sarcoma. Interestingly, both cancers utilize both telomerase and the alternative lengthening of telomeres mechanism which do not exhibit THOR hypermethylation [28–29]. Furthermore, the more indolent thyroid cancer exhibited the lowest levels of THOR hypermethylation.

### THOR distinguishes benign from malignant prostate tissue

In order to test whether the hypermethylation observed in the CG site within THOR in PCa is unique to this specific area of the *TERT* promoter we analyzed the methylation status of the whole *TERT* gene using MeDIP-seq on 51 PCa and 53 normal prostate tissues [30]. While methylation between normal prostate and PCa tissues is similar throughout the gene (Supplementary Figure S1), a significant difference in methylation between cancerous and normal tissue was observed only in the promoter region of *TERT* (*p* = 7.5 × 10^−12^), directly matching THOR (Figure 1B). To further explore THOR as a candidate cancer biomarker in PCa, we used pyrosequencing on multiple PCa samples from our discovery cohort. In 164 prostatectomies where matched benign and malignant tissues were available, THOR was significantly hypermethylated in the PCa component (*p* < 0.0001, Figure 1C).

### THOR Hypermethylation correlates with other bio-pathological risk factors

To further elaborate on THOR's association with known PCa risk factors, we initially compared THOR methylation with increasing Gleason scores. A significant difference between benign tissue and Gleason 6 PCa was observed (*p* < 0.0001). Analysis of THOR (as a continous variable) and Gleason revealed that THOR Hypermethylation is positively associated with Gleason score (X^2^ = 9.60; *p* = 0.0082). Although THOR methylation exhibited significant difference between low grade (Gleason 6) PCa and high grade (Gleason score ≥ 8) (*p* = 0.0416, Figure 1D) this was not observed between Gleason 6–7 tumors. We then determined the association between THOR and different established risk criteria. High-risk tumors (Gleason score ≥ 8 or PSA ≥ 20 ng/mL) had significantly higher THOR methylation than low risk PCa (Gleason score 6 and PSA < 10 ng/mL) in both the discovery cohort (*p* = 0.0436, Supplementary Figure S2A) and the validation cohort (*p* = 0.0397, Supplementary Figure S2B). Interestingly, higher THOR methylation was associated with locally advanced disease in both the discovery and validation cohorts (*p* = 0.0440 and *p* = 0.0147 respectively, Supplementary Figure S2C and S2D). In contrast, we didn't observe any association between THOR methylation and PSA levels, age, prostate volume or TMPRSS2-ERG gene fusion [31] (Supplementary Table S2). Margin status was also evaluated as a potential prognostic factor for biochemical relapse. However, this parameter was not significantly associated with time to biochemical relapse in both cohorts (Discovery cohort *p* = 0.2; Validation cohort *p* = 0.9).

### THOR as a novel risk stratification marker for low and intermediate Gleason PCa

**Figure 2:**
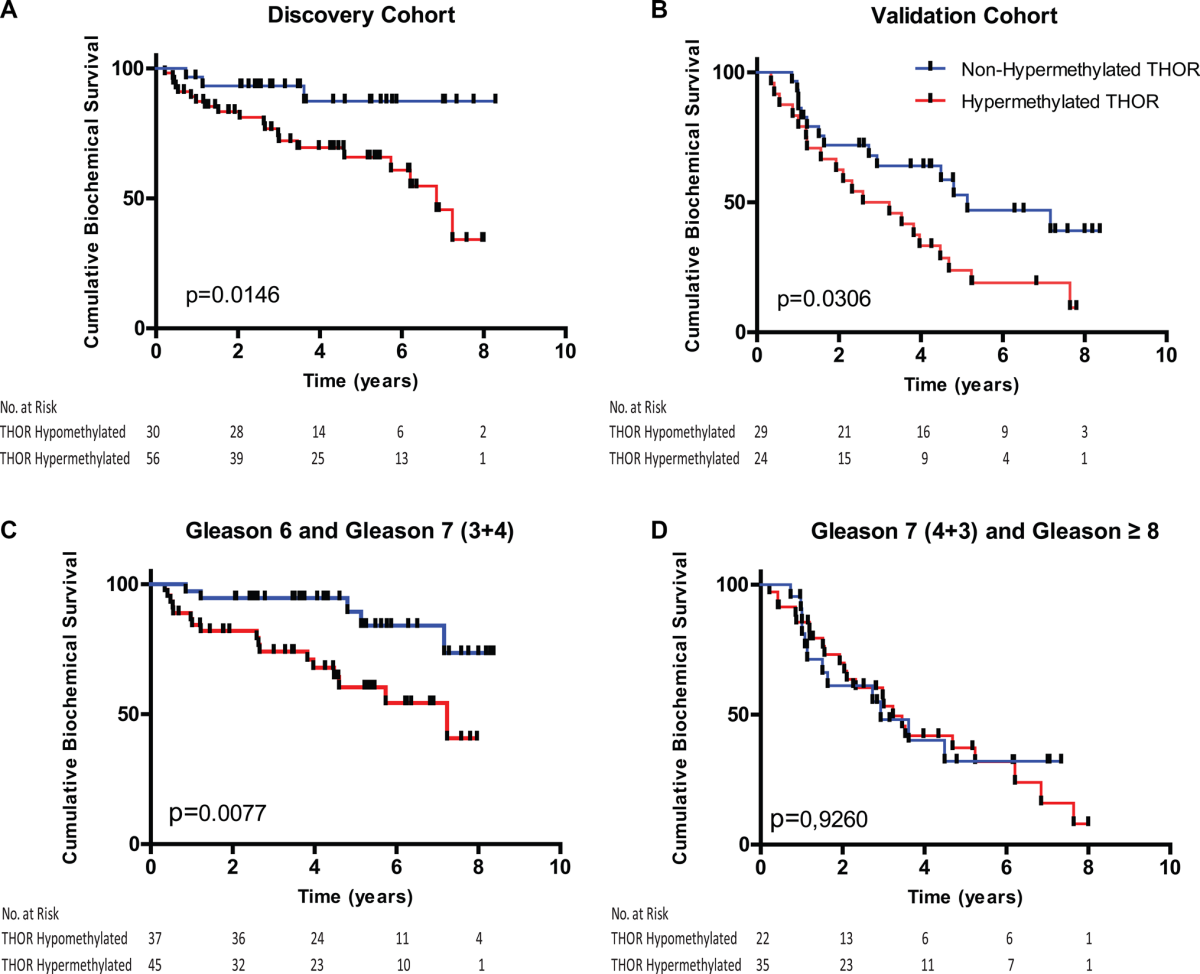
Levels of THOR methylation stratify prostate cancer patients Biochemical recurrence reveals that patients from all Gleason scores (6, 7 and ≥ 8) with low levels of THOR methylation show significantly better biochemical progression free survival in both the discovery (**A**) and validation cohorts (**B**). Patients from both cohorts with Gleason 6 and intermediate risk (Gleason 7 (3 + 4)) and low THOR status present significantly better progression free survival when compared to patients with high levels of THOR methylation (**C**). Considering higher risk groups (Gleason 7 (4 + 3) and Gleason ≥ 8 patients)) the levels of THOR methylation did not show differences in terms of biochemical progression free survival (**D**).

To assess the role of THOR as a prognostic marker in PCa, we chose a threshold of 20% methylation as previously done (AUC of 0.799, *p* < 0.0001) [27]. The median follow-up for patients in the discovery cohort was 9,8+/−3,8 years and for patients in the validation cohort was 7,9+/−2,5 years. A total of 139 patients were analyzed for biochemical relapse and THOR methylation. Five-year biochemical progression free survival (BPFS) for the discovery cohort was 65% (95% CI 52–78) and 87% (95% CI 73–100) for THOR hypermethylated and non-hypermethylated PCa respectively (*p* = 0.015, Figure 2A). Similar superior BPFS was observed for non-hypermethylated PCas in the validation cohort (*p* = 0.0306, Figure 2B). Combining the 2 cohorts revealed similar superior BPFS for non-hypermethylated PCa (*p* = 0.01, Supplementary Figure S3A). To determine the ability of THOR to predict BPFS in the clinically low and intermediate PCa, we analyzed patients with lower Gleason scores. THOR non-hypermethylation was associated with improved BPFS in both Gleason 6 and 7 PCa (*p* = 0.016 and *p* = 0.02 respectively, Supplementary Figure S3B and S3C). Since Gleason 7 tumors are known to have diverse clinical outcomes and have shown heterogeneous THOR values, we divided our survival analysis to Gleason 3 + 4 and 4 + 3 subgroups. Strikingly, THOR status predicted BPFS in Gleason 7 (3 + 4) tumors but failed to do so in Gleason 7 (4 + 3) PCa (Supplementary Figure S3D, S3E). Furthermore, most Gleason 8 PCa exhibited THOR hypermethylation and THOR values did not predict survival for higher Gleason tumors (Supplementary Figure S3F). Combined, THOR methylation defines a novel risk group for PCa where in lower Gleason 6 and 7 (3 + 4) THOR predicts outcome (*p* = 0.007) while this signature is lost in higher Gleason scores (Figure 2C and 2D). Univariate and multivariate analysis revealed that for this risk group (Gleason 6 and Gleason 7 (3 + 4) THOR Hypermethylation has the highest risk for recurrence with HR of 6.224 (*p* = 0.0012; C-índex 0.91, Table 1) and is an independent risk factor for BPFS (HR: 3.684; Ci: 1.81–11.5; *p* = 0.0247).

**Table 1:** Univariate and multivariate analysis of time to biochemical recurrence among patients with Gleason 6 and Gleason 7 (3 + 4)

	UNIVARIATE ANALYSIS				MULTIVARIATE ANALYSIS				
	HR	95% CI	Chi Square	*P*	C índex	HR	95% CI	Chi Square	*P*
***Both Cohorts***									
**Age**	1.047	0.972 to 1.172	1.4585	0.222	0.62	1.036	0.960 to 1.17	0.083	0.3623
**pT** (localized *vs* advanced)	2.748	1.176 to 6.469	5.4423	0.0197	0.88	2.454	0.967 to 6.230	3.685	0.0589
**PSA** Groups (< 10 *vs* ≥ 10)	3.288	1.307 to 8.271	6.3995	0.0114	0.92	2.549	0.931 to 6.979	3.314	0.0687
**THOR**	6.224	1.314 to 9.751	6.2240	**0.00126**	0.91	3.685	1.181 to 11.501	5.046	**0.0247**

### Combining PSA and THOR analysis significantly increases patients’ outcome prediction

**Figure 3:**
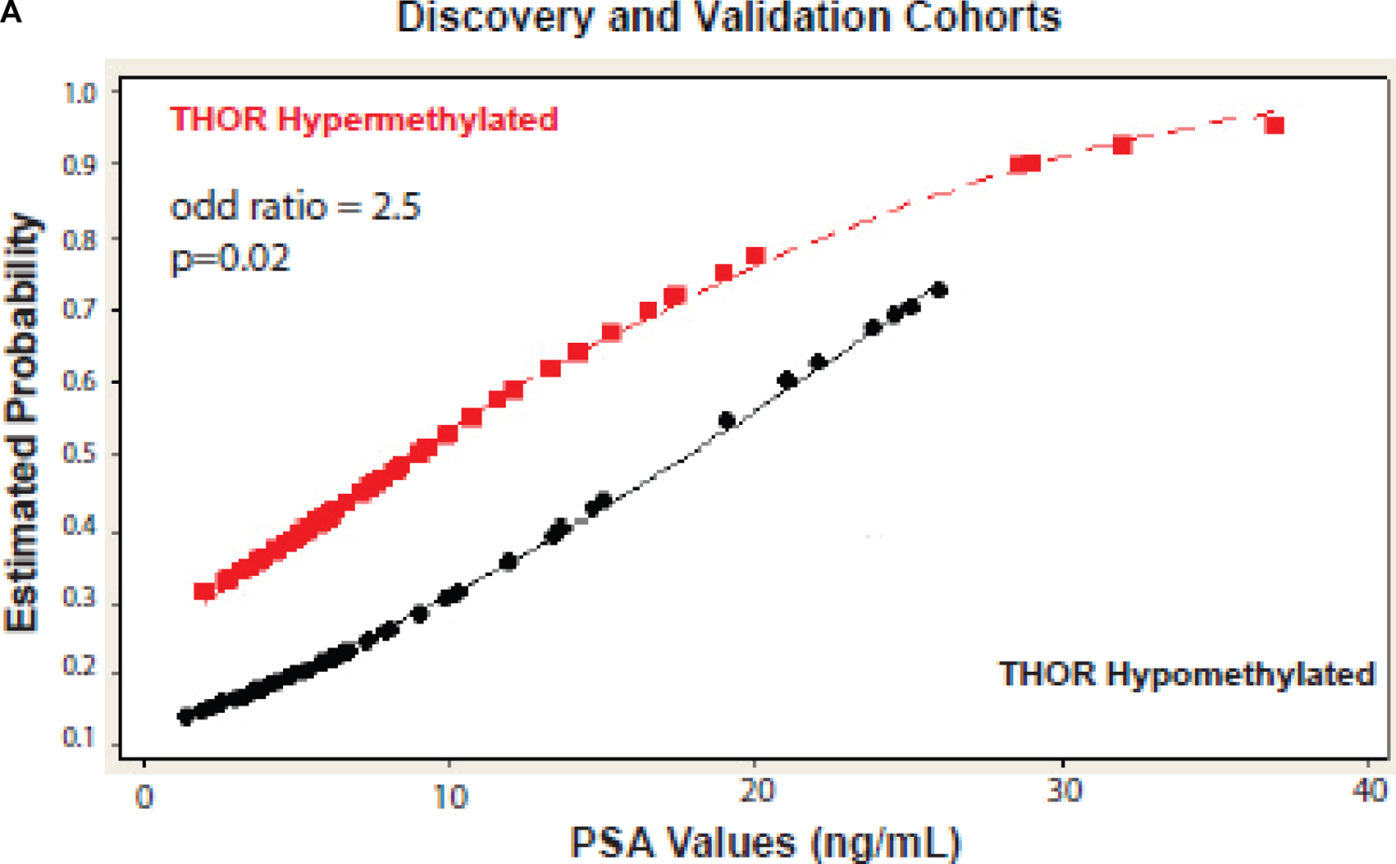
Estimated probability for biochemical relapse Analysis of both cohorts together reveals that patients with high levels of THOR methylation have a significant increase in the probability of recurrence for the same values of PSA when compared to patients with low levels of THOR methylation.

Finally, we tested the ability of THOR to add information to the commonly used PSA as a predictor of outcome. For each PSA value, THOR hypermethylation more than doubled the risk of BPFS (OR 2.5, *p* = 0.02, Ci: 1.15–5.6; Figure 3). These findings were highly consistent between cohorts (Supplementary Figure S4A and S4B). All PCa with very high PSA (> 25 ng / mL) were hypermethylated and > 90% experienced tumor recurrence.

## DISCUSSION

In this study we extend our previous observations, which suggest a clinical role for the methylation signature of the *TERT* promoter in cancer. Specifically, we show that THOR hypermethylation can serve as a robust and simple tool for predicting tumor behavior in low grade PCa, where management is controversial. These findings can add important information for the clinical management of these patients.

Global DNA methylation status is highly variable during early embryogenesis but is quite stable in most tissues throughout life [32–34]. Aberrant methylation of the cancer genome has major implications on gene expression and has been recently reported to refine tumor subgroups and be associated with patient survival [35, 36]. Since the assays performed are based on whole-genome arrays, changes observed are usually global and determination of methylation status is restricted to the whole gene or its promoter. Analysis of methylation regions within genes are rarely performed [37] and has not been developed as a tool for clinical diagnostics. Specifically, data on methylation status of regions within a promoter of an oncogene and its effect on gene expression in cancer are limited. Moreover, other alterations which result in telomere maintenance in cancer such as *TERT* promoter mutations and the presence of alternative lengthening of telomeres are rarely observed in PCa. [38–39]. Our observations suggest that mapping of *TERT* methylation (Supplementary Figure S1) can add valuable information to the mechanisms of oncogene activation during carcinogenesis. We have previously shown that THOR methylation is a dynamic process during gliomagenesis [27]. Further studies are required to understand the causes and consequences of hypermethylation of this specific region on *TERT* activation and PCa progression.

As in most advanced carcinomas, aggressive PCa express telomerase, and interestingly, for tumors with high Gleason scores (≥ 8) THOR lost its prognostic value. Although this is mainly due to the vast majority of these cancers having high THOR methylation and the small amount of tumors with low methylation values to show statistical difference, this further suggests that in contrast to early stages PCa, where some tumors might lack self-renewal capacity, advanced stage tumors will maintain their telomeres by either THOR hypermethylation or other pathways to activate telomerase.

Nevertheless, currently approximately 50% of newly diagnosed PCa patients are found to have a low risk prostate cancer [45] and a significant proportion of those cancers may never become life threatening. Furthermore, the risk of biochemical recurrence after definitive surgery is highly variable and is usually poorly understood [46–48].

For this challenging patient population, THOR analysis adds a new dimension for the decision making process:

First, among patients with Gleason 6 PCa, the risk of tumor recurrence is extremely low if THOR is non-hypermethylated (no progressions in 5 years, Supplementary Figure S3B) [49, 50]. This candidate biomarker may be highly valuable for patients undergoing active surveillance protocols, however, further confirmation from biopsy tissues will be required.

Second, the most relevant observation in our study is that THOR stratifies Gleason 7 tumors into 2 risk groups where the 3 + 4 group could be stratified with the Gleason 6 tumors while the 4 + 3 PCa are comparable with higher Gleason scores (Supplementary Figure S3) [51]. If confirmed, it will be possible in the future to lump the low (Gleason 6) and intermediate risk (Gleason 7 (3 + 4)) PCa to a single risk group for a more conservative management based on THOR status [52]. Indeed, for this combined group, THOR showed to be able to stratify patients into particularly low risk of recurrence (only 5% of patients with THOR non-hypermethylated PCa experienced biochemical recurrence within the first 5 years (Figure 2C)) while THOR hypermethylation increased the risk of recurrence by more than 6 fold (Table 1). This can be of clinical value for selecting patients to undergo adjuvant treatment after surgery and eventually applied to prostate biopsies establishing new criteria for active surveillance protocols.

Third, THOR status adds valuable information for each PSA level for these patients. THOR hypermethylated cancers have > 50% recurrence even with a PSA value of less than 10 ng/mL (Figure 3) while non-hypermethylated cancers carry a very low risk of recurrence in both cohorts.

Using telomerase as a biomarker has several advantages over some of potential biomarkers which have recently described for risk stratification of PCa [53, 54, 7]. Biomarkers which require RNA and use genomic information are complex, highly dependent on tissue quality and can only be performed in specialized laboratories. THOR is a DNA based marker and has proven in this and other studies to provide robust results in DNA from paraffin embedded samples and in degraded DNA. This assay could therefore be performed in most laboratories worldwide. Furthermore, telomerase represents a rational and attractive oncogene to pursue. Indeed, unlike other biomarkers, patients possessing this biomarker could have a potentially drug-targetable marker in that tumors with hypermethylated THOR could be treated using both telomerase inhibitors and demethylating enzymes.

Our studies have limitations related to retrospective cohorts. The use of biochemical relapse as an endpoint is suboptimal compared with prostate cancer-specific mortality or time to metastasis. Also, we acknowledge that our results are based on surgical specimens. However, analysis of these surgical specimens could identify within the low-intermediate risk group a subgroup of patients to whom early adjuvant treatment might be beneficial.

Furthermore, our data unveils the potential predictive value of THOR methylation when applied to tissue from prostate biopsies. THOR could stratify patients with low risk disease in both cohorts independently as the difference in outcome between cohorts did not change the general role of THOR as a candidate prognostic biomarker in PCa.

In the era of precision medicine where the aim is to determine patient-specific outcome, THOR hypermethylation represents a potential candidate biomarker for cancer diagnostics in biopsies. It can also determine aggressiveness of tumors with similar histological grade and be used as a companion biomarker for therapies using telomerase inhibitors or demethylating enzymes.

Finally, combining THOR with other biomarkers such as PSA can identify the patients with extremely low risk of recurrence where surveillance protocols could be applied. Further studies are required to verify if THOR methylation can change the current treatment paradigms in low or intermediate risk PCa.

## MATERIALS AND METHODS

### Patients

Tissue samples and patient data were obtained upon consent according to the Research Ethics Boards at the participating institutions. For the MeDIP-Seq analysis of *TERT*, tumors selected were staged pT2–pT4 and had Gleason scores ranging from 6 to 9 (Heidelberg, Germany) [30]. Two additional cohorts were definided to retrospectively study the clinical implications of THOR in PCa. These included a discovery cohort of patients from Austria (*n* = 164) and a separate validation cohort from Portugal (*n* = 103), submitted to radical prostatectomy and from which FFPE tissue was available. Patients were selected based on the availability of FFPE tissue, follow-up time and available clinical information. Gleason score classification was considered for patient selection in validation cohort (mimicking discovery cohort). Clinical outcomes were blinded at patient selection. Patients lost for follow-up or with missing values were not considered for outcome analysis. Clinical outcomes, PSA values and prostate biopsy results were not used as selection criteria.

Demographic analysis and clinical characteristics for the discovery and validation cohorts are described in Table 2. From each surgical specimen we analyzed the malignant and benign tissue separately. Pathological evaluation was performed by experienced uro-pathologists in both centres. Dominant lesions (higher Gleason score) were selected for this study, by macrodissection (where at least 70% of malignant cells were found). Benign tissue was isolated from the same surgical specimen where tumours were isolated.

**Table 2:** Demographic and clinical characteristics of discovery and validation cohorts

	Discovery Cohort (*N* = 164)			Validation Cohort (*N* = 103)	
		*n°* patients	%	*n°* patients	%
**Age, years**		60.2			
Mean (Min-Max)		6.7		62.7	
Stand Dev				6.1	
**PSA (ng/mL)**		**6.67**			
Mean (Min-Max)				**11.47**	
PSA < 10		141	87.6%	47	54.7%
10 ≥ PSA <2 0		14	8.7%	26	30.2%
PSA ≥ 20		6	3.7%	13	15.1%
***Total***		**161**		**86**	
**Gleason Score**					
***6***		50	30.5%	29	28.2%
***7***		76	46.3%	48	46.6%
***≥ 8***		38	23.2%	26	25.2%
***Total***		**164**		**103**	
**TNM**					
**Localized Disease**		**111**		**45**	
	pT2a	9	8.1%	8	17.8%
	pT2b	12	10.8%	7	15.6%
	pT2c	90	81.1%	30	66.7%
**Locally Advanced**		**53**		**58**	
	pT3a	35	66%	41	70.7%
	pT3b	14	26.4%	15	25.8%
	pT4	4	7.6%	2	3.5%
**Prostate Volume (cc)**					
Mean		30			51.94
Stand Dev		13.92			19.63
**Erg Gene Fusion**					
Present		**90**			
	G6	29	31.8%		
	G7	45	49.5%		
	G8 & G9	17	18.7%		
Absent		**57**			
	G6	19	33.3%		
	G7	20	35.1%		
	G8 & G9	18	31.6%		
Unkown		**17**			
**Erg Gene Fusion**					
Mean time for BPS (Years)		2.6		2.65	
*N°* of Events		24		33	

Biochemical Progression free survival (BPFS) was defined as the time from surgery to biochemical recurrence (PSA > 0, 2 ng/mL in two different measurements after an undetectable post-surgery PSA measurement). All patients submitted to any adjuvant treatment that could alter the normal evolution of the disease were excluded from outcome analysis.

### Open access data

Dataset from The Cancer Genome Atlas was extracted for the Illumina Infinium 450 k array beta values. Mean and standard deviation were calculated from beta values for each tumour type. The single probe located in the THOR region (cg11625005) was used for this analysis.

### MeDIP-Seq analysis

For in-depth analysis of the *TERT* gene methylation status MeDIP-Seq was used [30]. Library preparation 2.5 μg of genomic DNA from 51 prostate cancers or 53 normal prostate tissues were fragmented to 100 to 200 bp using the Covaris S2 system and end repaired with End Repair mix (Enzymatics) followed by a purification step (Qiagen DNA Purification Kit) and ligation of barcoded SOLiD sequencing adapters as previously described [30].

### Analysis of THOR methylation

Quantitative sodium bisulfite pyrosequencing was performed for THOR as previously described [27]. In brief, targeted assays were designed using the PyroMark Assay Design Software 1.0 (Qiagen). Forward ATGATGTGGAGGTTTTGGGAATAG, reverse CCCAACCTAAAAACAACCCTAAAT and sequencing GGAGGTTTTGGGAATAG primers were used for PCR and pyrosequencing. The assay target region was 36 bp in length comprising 5 CpG sites. In our assay < 5% of the samples failed pyrosequencing analysis. Calculation of the % of THOR methylation was done as a mean value of these sites as previously described [27]. For clinical correlative studies we used the cut-off of 20% methylation with an AUC of 0.799 (*p* < 0.0001).

### Statistical analysis

MeDIP-Seq statistical analysis was conducted using R (version 2.9.2). Read counts in 500 bp non-overlapping consecutive bins were normalised to sample wise read counts (reads per million). To assess the difference in THOR Hypermethylation between normal and malignant tissue a two-tailed Student7s *t* test was used. To test the association of THOR with Gleason Scores, localized prostate cancer disease and locally advanced disease Mann-Whitney *U* test was used. *P* Pearson test assessed relation between methylation and age and methylation and prostate volume. For the prognostic model we initially dichotomized into high and low-methylation for THOR groups by receiver operating characteristic (ROC) analysis. Biochemical progression free survival (BPFS) was determined by Kaplan-Meier Survival curves on 139 patients for whom all clinical information was available and were not excluded by our criteria. Patients not having experienced PSA recurrence were censored at their last PSA measurement. The proportional hazards assumption was verified by the log-negative-log survival distribution function for all variables. Univariate and multivariate Cox Proportional Hazards (CPH) regression analyses and log-rank tests were conducted. Both analyses (Univariate and Multivariate) were done for time for biochemical recurrence. To evaluate the prognostic strength of THOR, the C-index was used. To estimate the additional role of THOR methylation in predicting BPFS for patients with different values of PSA a logistic regression was defined. All statistical analyses were performed using SAS V9.3 (SAS, Cary, NC).

## SUPPLEMENTARY MATERIALS FIGURES AND TABLES


