# Supplementary material for: A cancer specific hypermethylation signature of the TERT promoter predicts biochemical relapse in prostate cancer: a retrospective cohort study
Source: Oncotarget. 2016 Jul 16;7(36):57726–36. doi: 10.18632/oncotarget.10639 (PMC5295385; doi:10.18632/oncotarget.10639)
Supplement: Supplementary file 1 [file oncotarget-07-57726-s001.pdf]

A cancer specific hypermethylation signature of the TERT promoter predicts biochemical relapse in prostate cancer: a retrospective cohort study

Supplementary Materials

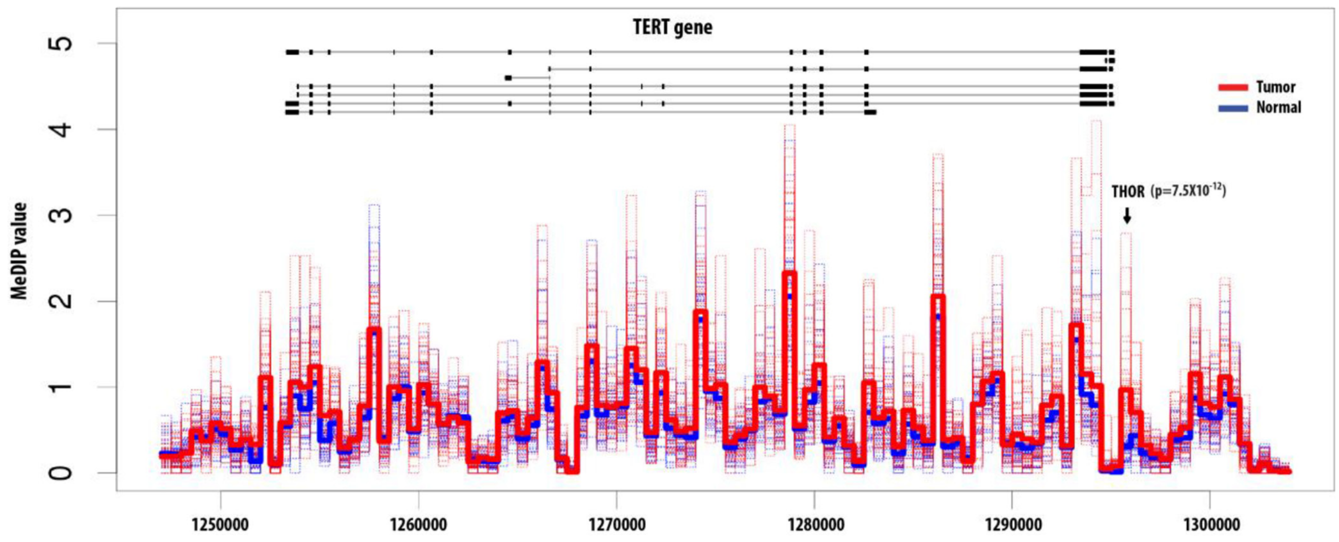

Supplementary Figure S1: Methylation status of the *TERT* gene. Methylation values of 51 tumours (dotted red lines) and their average (thick red line) as well as the methylation values of 53 normal prostate samples and their average (blue) are shown for 10000 bp wide regions in the *TERT* gene through MeDIP-seq analysis.

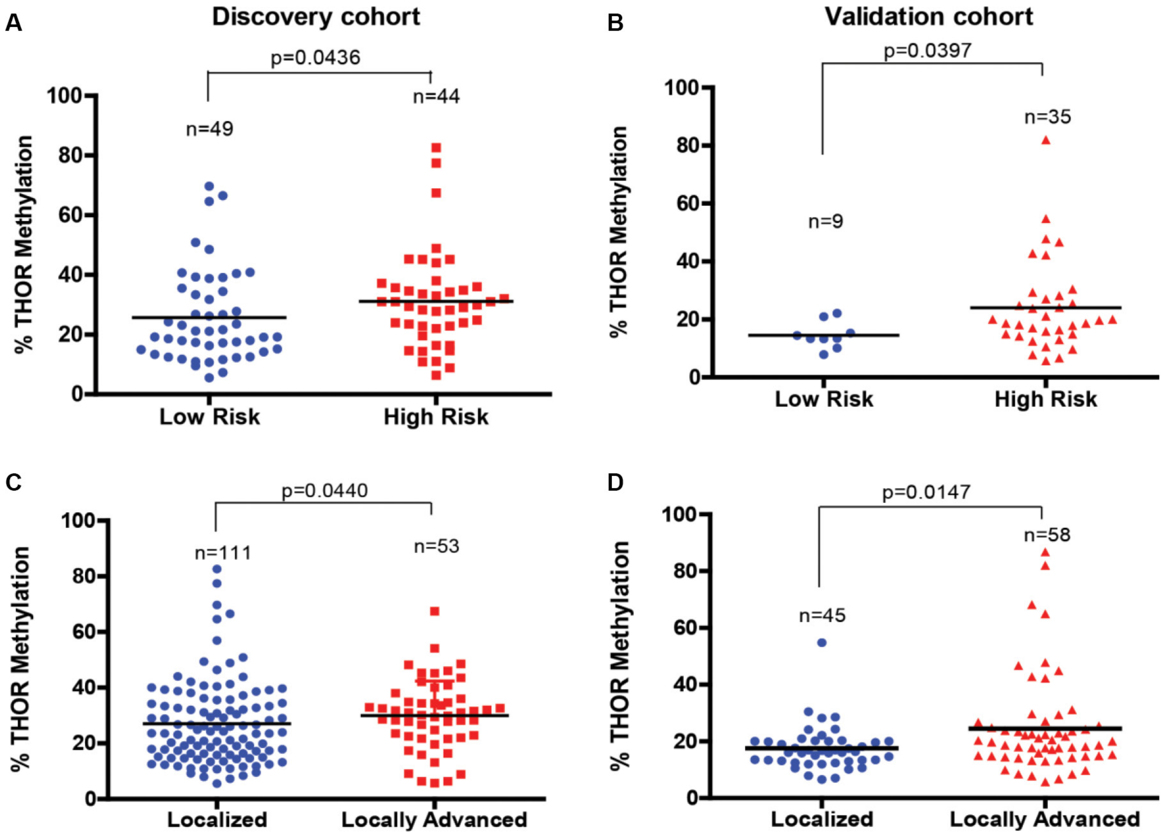

Supplementary Figure S2: THOR methylation associates wth high grade and invasive disease. Pyrosequencing analysis shows significantly higher levels of THOR methylation in patients with high-risk disease both in the discovery (**A**) and validation cohorts (**B**). Localized disease presents significantly lower levels of THOR when compared to locally advanced stages in the discovery (**C**) and validation (**D**) cohorts.

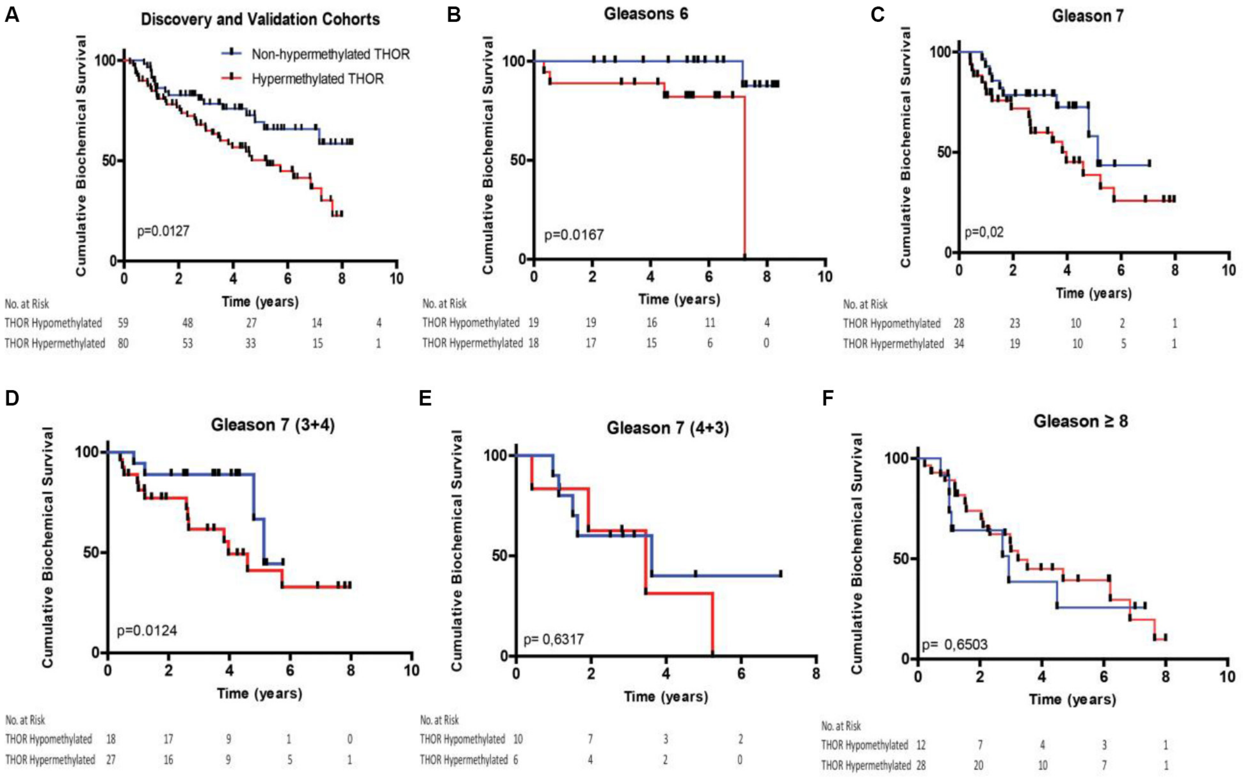

Supplementary Figure S3: Levels of THOR methylation stratify lower risk prostate cancer patients. (**A**) Biochemical recurrence reveals that patients from all Gleason scores and from both cohorts with low levels of THOR methylation show significantly better biochemical progression free survival when compared to patients with high levels of THOR methylation. (**B**) Gleason 6 patients are stratified by levels of THOR methylation. (**C**) THOR is not a significant marker to predict BPFS for intermediate Gleason 7 group at 8 years. (**D**) Gleason 7 (3 + 4) patients show a distinctive patter of BPFS at 5 years when stratified by levels of THOR methylation. THOR doesn't show significant predictor value for patients with higher Gleason scores 7 (4 + 3) (**E**) and ≥ 8 (**F**).

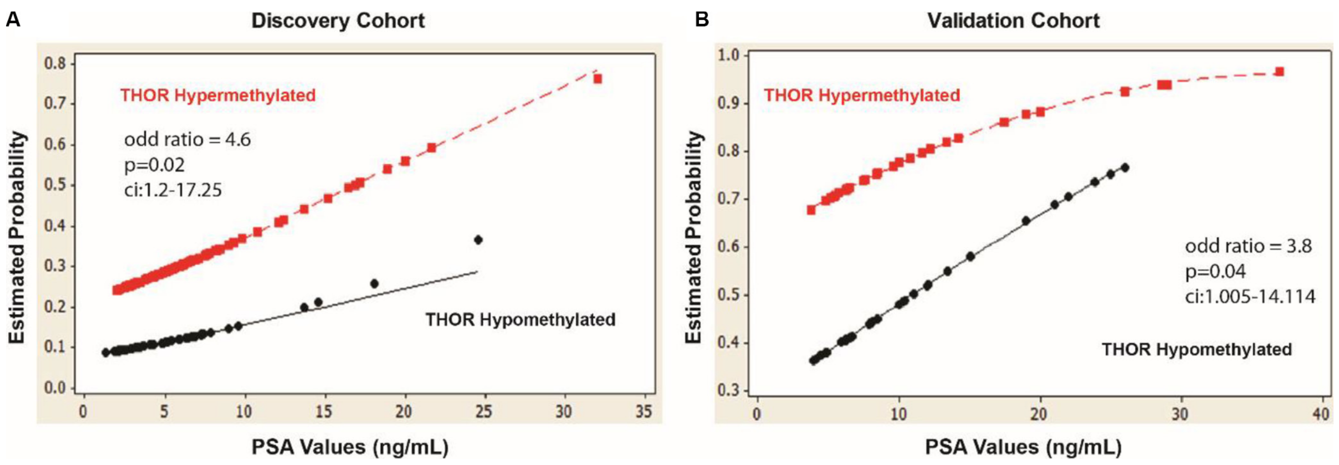

Supplementary Figure S4: Patients with higher levels of THOR have increased probability of recurrence. A significant increase in the probability of recurrence for the same values of PSA is observed in patients with high levels of THOR methylation in all samples from both the discovery (**A**) and validation cohorts (**B**).

Supplementary Table S1: Sample sizes for tumor tissues in TCGA pan-cancer cohort

Tumour Type	Total
Acute Myeloid Leukemia	194
Adrenocortical Cancer	80
Bladder Urothelial Carcinoma	242
Cervical and Endocervical Cancer	179
Colon Adenocarcinoma	275
Esophageal Carcinoma	73
Glioblastoma Multiforme	136
Kidney Papillary Cell Carcinoma	156
Brain Lower Grade Glioma	408
Prostate Adenocarcinoma	300
Sarcoma	129
Skin Cutaneous Melanoma	376
Thyroid Carcinoma	508

Supplementary Table S2: Correlation between THOR – Age, THOR –PSA

Discovery Cohort		Validation Cohort	
**Age**	*n* = 164	**Age**	*n* = 103
Pearson Test	*r* = 0.01	Pearson Test	*r* = 0.05
P value	0.83 (NS)	*P* value	0.59 (NS)
**PSA**	*n* = 164	**PSA**	*n* = 98
Pearson Test	*r* = 0.01	Pearson Test	*r* = 0.004
P value	0.83 (NS)	*P* value	0.96 (NS)
**Prostate Volume**	*n* = 153	**Prostate Volume**	*n* = 101
Pearson Test	*r* = 0.01	Pearson Test	*r* = 0.042
P value	0.83 (NS)	*P* value	0.67 (NS)
TMPRSS2-ERG	*n* = 148		
Mann-Withney			
P Value	0.737		

THOR- Prostate volume and THOR – *ERG* gene.
